# Biomedical drugs and traditional treatment in care seeking pathways for adults with epilepsy in Masindi district, Western Uganda: a household survey

**DOI:** 10.1186/s12913-019-4879-2

**Published:** 2020-01-06

**Authors:** Elizeus Rutebemberwa, Charles Ssemugabo, Raymond Tweheyo, John Turyagaruka, George William Pariyo

**Affiliations:** 1 Department of Health Policy, Planning and Management, Makerere University School of Public Health, Kampala, Uganda; 2 African Centre for Health and Environmental Studies, Kampala, Uganda; 3 Department of Disease Control and Environmental Sciences, Makerere University School of Public Health, Kampala, Uganda; 4 Department of Public Health, Lira University, Lira, Uganda; 5 District Health Office, Masindi District Local Government, Masindi, Uganda; 6 Johns Hopkins Bloomberg School of Public Health, Department of International Health, Baltimore, MD USA

**Keywords:** Epilepsy, Care seeking, Pathway, Biomedical drugs, Traditional herbs, Prayers, Uganda

## Abstract

**Background:**

Many patients with epilepsy in sub-Saharan Africa do not receive adequate treatment. The purpose of the study was to identify the health care providers where patients with epilepsy sought care and what treatment they received.

**Methods:**

A cross sectional study was conducted across 87 out of 312 villages in Masindi district. A total of 305 households having patients with epilepsy were surveyed using an interviewer administered questionnaire. Data was entered and analysed in Epi-info ver 7 for univariate and bivariate analysis, and in Stata SE ver 15.0 for multivariable analysis. Sequences of health providers consulted in care seeking, rationale and drugs used, and factors associated with choice of provider were assessed.

**Results:**

A total of 139 out of 305 (45.6%) households offered some treatment regimen at home when patients got symptoms of epilepsy with 44.6% (62/139) giving herbs and 18.0% (25/139) offering prayers. Eight different types of providers were consulted as first contact providers for treatment of epilepsy. Health centres received the highest percentage 35.4% (108/305) followed by hospitals 20.9% (64/305). A total of 192 of 305 (63.0%) households received anti-epileptic drugs, 13.1% (40/305) received prayers and 21.6% (66/305) received herbs at the first contact care seeking. Compared to a health centre as the first choice provider, other facilities more significantly visited were; hospitals if they were perceived as nearer (adj. Coeff 2.16, 95%CI 0.74, 3.59, *p* = 0.003), churches / mosques if cure for epilepsy was expected (adj. Coeff 1.91, 95%CI 0.38, 3.48, *p* = 0.014), and traditional healer for those aged ≥46 years (adj. Coeff 5.83, 95%CI 0.67, 10.99, *p* = 0.027), and friends/neighbour for traders (adj. Coeff 2.87, 95%CI 0.71, 5.04, *p* = 0.009).

**Conclusion:**

Patients with epilepsy seek treatment from multiple providers with the public sector attending to the biggest proportion of patients. Engaging the private sector and community health workers, conducting community outreaches and community sensitization with messages tailored for audiences including the young, older epileptics, traditional healers as stakeholders, and traders could increase access to appropriate treatment for epilepsy.

## Background

It is estimated that over 50 million people have epilepsy worldwide and nearly 80% of these live in low and middle income countries. In sub-Saharan Africa, about 60% of the patients do not receive treatment due to social or economic reasons [1]. The treatment gap for epilepsy in Uganda has been estimated at 78% [2].

Various studies have highlighted the use of traditional medicine in addition to modern medicine in Uganda [2–4]. Care seeking for chronic illnesses like epilepsy takes a long time and patients pass through various providers. Information on the different providers through whom the patients pass and the treatment options they receive is critical to developing strategies to increase coverage of epilepsy patients receiving effective evidence-based modern treatments and remedies.

Uganda is estimated to have prevalence of epilepsy ranging between 2.2 and 12.6 per 1000 population [1]. There is ample literature demonstrating inadequate treatment for persons with epilepsy [2, 5–7]. However, there is not much literature on the sequence of care seeking, what drives patients to change from one provider to another and where most of the patients terminate their care seeking. From a health system’s perspective, the pathways of care seeking for a chronic condition such as epilepsy, and the determinants of such choices are important attributes for organising a healthcare system across multiple providers (public, private, and public-private partnerships) that is responsive, and guarantees continuity of care for repeat presentations. The objective of this study was to assess the providers through whom the patients with epilepsy in Masindi district pass as they seek care, where they terminate their care seeking and the remedies offered from these various providers.

## Methods

### Study site

The study was conducted in Masindi district located in Western Uganda 216 Kilometers away from Kampala. Masindi district is at an average altitude of 1295 m above sea level situated between latitude 1^0^ 22′ and 2^0^ 20′ North of the Equator, and longitude 31^0^ 22′ and 32^0^ 23′ East of Greenwich [8]. The district has three health sub-districts of Bujenje, Buruli and Masindi municipality. It has 23 government health facilities including one district hospital (about 100 beds), one Health Centre level IV (equivalent to a small rural hospital), six Health Centre level III facilities (basic preventative, outpatient curative and maternity services) and 15 Health Centre level II facilities (basic outpatient and preventative services) [9]. According to the Uganda National Population Census report of 2016, Masindi district had about 290,000 people in 2014 [10]. Masindi is one of the epilepsy high-burdened districts in Uganda as identified by the non-communicable disease (NCD) & neglected tropical diseases desk at the National Ministry of Health.

### Study population

The study population consisted of households with a patient presenting with epileptic seizures identified by a community health worker of that village. Regardless of whether there was one or more patients with epileptic seizures in one household, to enhance the possibility of recall, only care seeking for the most recent epileptic episode was assessed. The household head was the primary respondent, but if absent, another responsible adult was interviewed.

### Sampling and accessing study participants

In order to select the study households, the district health office was requested to give a list of the villages which, according to their health management information system records, had registered patients with epilepsy. Out of the 312 villages in the district, the district health office was able to provide 87 villages located in 25 of the 32 parishes, and spread across all the 9 sub-counties in Masindi District. All these villages where a case of epilepsy had been identified in the district health database were covered by this survey.

The District Health Office offered the services of the community liaison focal person to support the research assistants to ensure a smooth introduction to communities to carry out data collection. Community health workers were told to be ready to meet the research assistants on specific days. The district community liaison officer accompanied the research assistants and introduced them to the community health workers who then took and introduced the enumerators to the households which had been identified as having patients with epilepsy.

### Data collection

Six research assistants collected data in January and February 2019. They were all experienced in population based survey data collection. Prior to commencing field work they were trained by the investigators for two days on the objectives of the study, key issues to consider while collecting quantitative data, the questionnaire they were to use and refreshed on ethical conduct in research data collection and interactions with respondents. During the training, they did role plays to ensure that they grasped the details. The tool used in the survey was adopted from one used in an earlier study by the lead investigator which assessed the pathways in diabetic care seeking [11]. The pathways to diabetic care seeking tool was found valid in assessing care pathways for a chronic condition, and was deemed the most suitable, given that none existed for epilepsy within the study’s geographical region. The coded tool was independently translated into the local language (Runyoro) by a translator, and back translated separately into English. Consistency of the back-translated version of the questionnaire was assessed during the training of research assistants, who were all fluent in Runyoro, the local language for the survey. All open ended responses were translated into English by one of the data entrants, who was independent of the data collectors.

The interview and data collection at the respondent’s residence took place either within the house or in a suitable open space outside the main house, taking precautions to ensure privacy during the interview.

The investigators closely supervised the research assistants during data collection in the field, answered questions and addressed any issues that needed clarification. Every evening, the research assistants and the investigators met and discussed the progress of data collection for the day, reviewed challenges faced and planned the data collection of the next day.

### Data management

The filled questionnaires were checked for completeness in the field by the study investigators. The investigators developed the data entry screen which was given to a data entrant who entered all the data into a personal computer. A 10% sample of the questionnaires were cross checked and verified for accuracy of data entry. All the questionnaires were entered and analysed using Epi info 7 (Centers for Disease Control and Prevention, Atlanta, Georgia). The Epi info 7 dataset (dataset.prj) was exported to Stata SE version 15 (dataset.mdb), where multinomial logistic regression analysis were conducted.

### Data analysis

Data was analysed for descriptive statistics of socio-demographics such as age, sex, marital status, occupation, and educational attainment. Additional descriptive statistics were computed for key variables such as the sequence of choice of provider, type of care received at each provider, perceptions of care received and reasons for termination of care at each provider. The pathways through which households sought care were generated by cross tabulation of where households went for the care seeking at consecutive times, for instance, to compute how households moved from the first provider to the second, the place where households sought care for the first time was tabulated against where they sought care for the second time. This generated proportions of households with patients who came from one provider type and went to each of the other respective types of providers at the next care seeking contact.

To analyse the treatment received by those who gave some treatment at home and those who reported having not given any treatment, a sub-analysis was done for the respondents who indicated having given herbs and prayers at home separately from those who reported having done nothing.

A bivariable multinomial logistic regression analysis was conducted for the outcome of first-choice of provider, and the plausible independent variables. The outcome variable was polychotomous and used the nominal scale including categories such health centre, hospital, church/mosque, friend/ neighbour and traditional healer and other. The Independent variables included the socio-demographic characteristics, reason for shifting provider as the main independent variable, and others including type of treatment received, and perceptions of treatment. All the significant variables in the bivariate analysis at *p* < 0.05 and all plausible independent variables were introduced consecutively into a multivariable multinomial logistic regression analysis to obtain corresponding adjusted logistic coefficients, 95% confidence intervals and *p*-values, for the various categories of the outcomes. A Hesemer-Lemeshow goodness-of-fit test was conducted to confirm the best model.

### Ethical considerations

This study was approved by the Makerere University Higher Degrees and Research and Ethics Committee (642) and the Uganda National Council for Science and Technology (HS 4867). Permission was got from Masindi district local government and from community leaders in the villages where data was collected. Informed written consent was obtained from all the respondents. Participants remained with a copy of the consent form. The data was secured and only accessed by the research team.

## Results

### Descriptive statistics

#### Characteristics of respondents

A total of 305 respondents were interviewed from 87 villages in 25 parishes cutting across all the nine sub-counties of the district. Table 1 shows the socio-demographic characteristics of the participants.

**Table 1:** Socio-demographic characteristics of respondents

Variables	Freq (%)
Position in the household	
Children	28 (09.18)
Household head	172 (56.39)
Relative	18 (5.90)
Wife	87 (28.52)
Age in completed years	
18–25	32 (10.49)
26–35	70 (22.95)
36–45	66 (18.86)
46–55	66 (18.86)
56–65	37 (12.13)
> 65	34 (11.15)
Sex	
Female	184 (60.33)
Male	121 (39.67)
Marital status	
Married/cohabiting	190 (62.30)
Separated/Divorced	44 (14.43)
Single/Never married	31 (10.16)
Widow/Widower	40 (13.11)
Highest level of education attended	
None	61 (20.00)
Primary	179 (58.69)
Secondary	51 (16.72)
Tertiary (Others)	12 (03.93)
Tertiary (University)	2 (00.66)
Occupation	
Farming	231 (75.74)
Government worker	12 (03.93)
Trading	36 (11.80)
Unemployed	11 (3.61)
Others: artisans, casual labourers, boda boda riders etc.	15 (4.92)

All the respondents were members of the household. The majority were household heads, others were spouses, children aged 18 years or older and a few were close relatives. The majority of the respondents were females. Only about 20% did not have any formal education. About three quarters of the respondents were farmers.

All of the 305 respondents interviewed provided valid responses (*n* = 305) on the most common signs and symptoms observed in the most recent episode of an epileptic attack. Fits and seizures presented in 71.5% of the patients. Other symptoms were unconsciousness which presented in 30.8%, severe fever in 23.0%, severe headache in 18.7%, shivering and shaking in 15.7%, shouting in 14.1%, body weakness in 12.5%, uncontrolled urination in 11.8% and red eyes in 11.5%. Other symptoms were ‘being unsettled’ which presented in 8.2% and oversleeping in 6.9%. Low concentration and dizziness both presented each in 6.6%. Others were being absent minded that presented in 5.9%, talking a lot in 5.6%, being moody in 5.3%, while the rest of the symptoms like decreasing appetite, biting teeth, increased appetite, uncontrolled defecation and depression each presented in less than 5% of the patients.

#### Transitioning from one provider to another

**Fig. 1:**
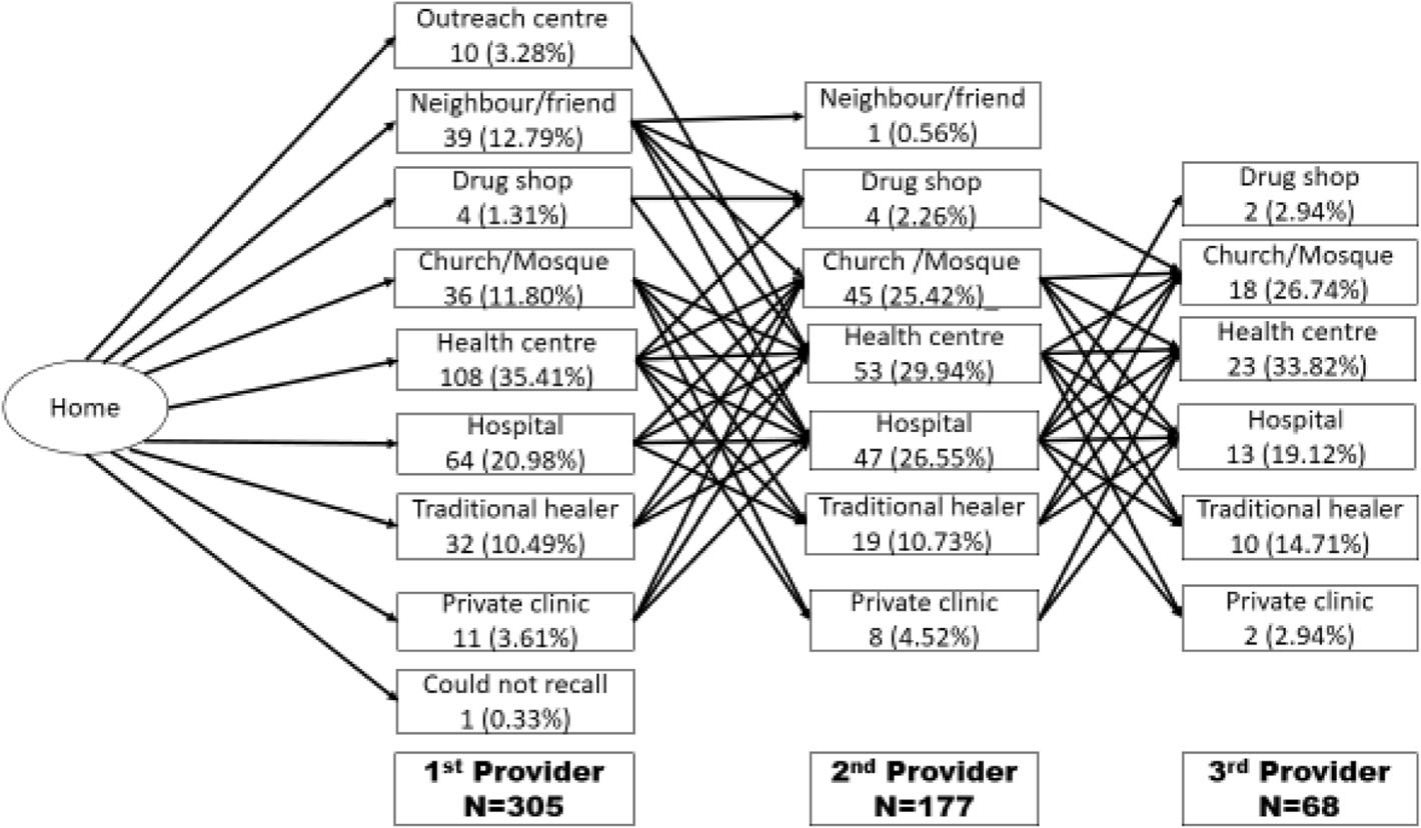
Care seeking from home through the first three providers

All the 305 households visited indicated that they had sought treatment for the last episode of epilepsy outside of the household. Whereas some stopped at one provider, 13 respondents (4.3%) reported visiting up to a fourth provider and two respondents reported seeking care from up to five consecutive providers. The numbers that went to each of the providers as first, second or third provider are illustrated in Fig. 1.

Five types of providers were each reported to have been first point of care by more than 10% of respondents (*n* = 305) namely; health centre 35.4%, hospital 21.0%, neighbour/friend 12.8%, church/mosque 11.8% and traditional healers 10.5%. For the second and third contact provider, only four provider types were reported to have continued caring for more than 10% of the patients with epilepsy that sought care. These were the health centres, hospitals, church/mosque and traditional healers. Overall, the health centres saw the majority of epilepsy patients, about one-third of the total at each of the first, second and third visits.

#### Reasons for shifting from one provider to another

Respondents were asked why they shifted from one provider to another. This was an open ended question. At analysis, the responses were categorized and summarized as shown in Table 2.

**Table 2:** Reasons for shifting from one provider to another

Variables	Frequencies at each shifting			
	From 1st to 2nd Provider *n* = 177 (%)		From 2nd to 3rd provider, *n* = 68 (%)	From 3rd to 4th provider, *n* = 13 (%)
Reason for shifting				
Did not get better hence sought better treatment	125 (70.6)		49 (72.1)	08 (61.5)
New provider was nearer	22 (12.4)		13 (19.1)	02 (15.4)
Advice from friend or neighbour	12 (6.8)		02 (2.9)	03 (23.1)
God needs to bless the drugs	11 (6.2)		02 (2.9)	00 (0.0)
Expenses were high at former provider	02 (1.1)		01 (1.5)	00 (0.0)
Drugs were free at new provider	01 (0.6)		00 (0.0)	00 (0.0)
Other^a^	04 (2.3)		01 (1.5)	00 (0.0)

Table legend ^a^includes the herbalist shifting, the patients being taken by the mother or wanting to know the disease

Most of the respondents indicated that they shifted from one provider to another because they had not improved and they were looking for better treatment. A few others reported going to providers who were nearer or being advised by friends or neighbours to seek treatment from other providers.

Respondents were asked the purpose of seeking treatment, whether it was for controlling fits and to continue living with epilepsy or for getting a cure for epilepsy. The results are shown in Table 3. More than half of those who sought care from the first and second provider wanted a cure for epilepsy. It is only at the third and fourth provider that over half of the respondents aimed at controlling their fits.

**Table 3:** Sequence of provider visited, and care received for epilepsy

Variables	1st Provider visit, *n* = 305 (%)	2nd provider visit, *n* = 177 (%)	3rd provider visit, *n* = 68 (%)
Health centre	108 (35.4)	53 (29.9)	23 (33.8)
Hospital	64 (20.9)	47 (26.6)	13 (19.1)
Church/Mosque	36 (11.8)	45 (25.4)	18 (26.5)
Traditional healer	32 (10.5)	19 (10.7)	10 (14.7)
Neighbour/friend	39 (12.8)	01 (1.6)	00 (00.0)
Other*	26 (08.5)	12 (6.7)	04 (05.8)
Control fits/seizures	111 (36.4)	74 (41.8)	40 (58.8)
Cure epilepsy	194 (63.6)	103 (58.2)	28 (41.2)
Anti-epileptic medicines	192 (62.9)	113 (63.8)	40 (58.8)
Herbal medication	66 (21.6)	19 (10.7)	10 (14.7)
Prayers	40 (13.1)	45 (25.4)	18 (26.5)
Others^&^	07 (02.3)	00 (00.0)	00 (00.0)

*other includes private clinics, drug shops, and community outreach

^&^ Others include doing nothing

#### Providers where seeking care was terminated

About 42.0% (128/305) of the respondents just sought care from one provider, while 61.6% (109/177) and 80.9% (55/68) terminated care at their second and third provider respectively. Only 13 patients sought care from a fourth provider and two from a fifth provider. Considering all the patients (*n* = 305), 36.0% terminated their care at health centres, 23.3% terminated it at a hospital, 17.7% at a church/mosque, 10.5% at a traditional healer, 3.6% at a private clinic, 3.0% at a neighbour/friend, 3.0% at a drug shop, and one respondent (0.33%) could not recall.

The health centre level was the predominant provider of care for the patients with epilepsy followed by hospitals. However, the church/mosque and traditional healers each maintained a significant presence of more than 10% of patients seen at first, second or third provider contact level.

#### Treatment received by patients with epilepsy

Of all the 305 respondents, about half, 54.4% (166/305) reported to have not given any treatment at home when the patient last presented with signs and symptoms of epilepsy. The remaining 45.6% (139/305) offered different treatments with 44.6% (62/139) reporting having given local herbs, 18.0% (25/139) offering prayers, 15.1% (21/139) giving antimalarial treatment like Coartem and Chloroquine, and 7.2% (10/139) providing a pain killer (Paracetamol). The rest of the remedies offered at home were each given by less than 5% of the respondents and they included putting the patient under a tree, giving the patient something to drink, giving a medication with sedating effects (e.g., Chlorpheniramine tablets) or offering blessed or ‘holy’ water from the church.

**Fig. 2:**
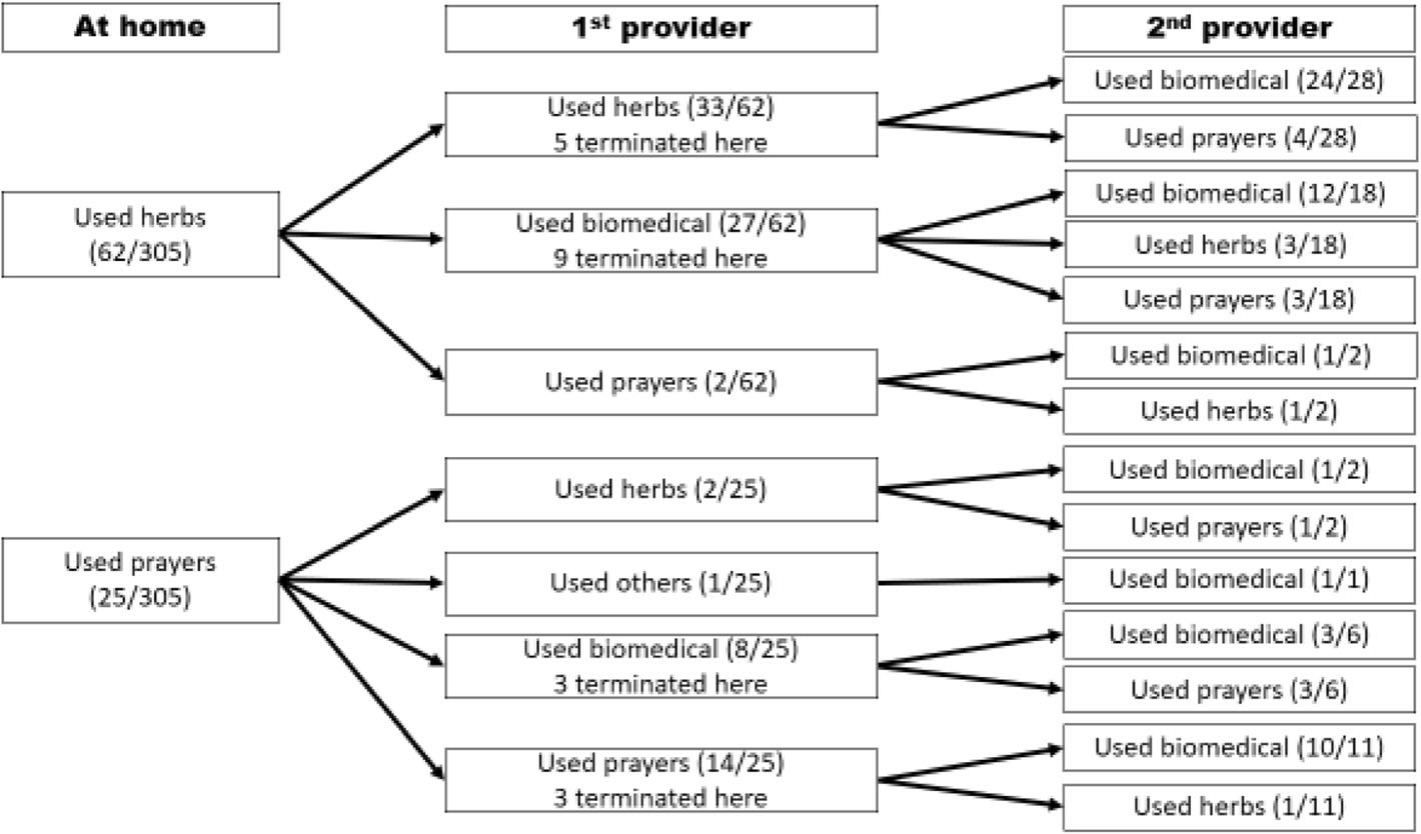
Treatment received by patients at first and second provider who had received herbs or prayers at home

A sub-analysis of those households reporting having first offered herbs or prayers at home before seeking care outside the home to a first and second contact provider is shown in Fig. 2.

It can be noted that even up to the second provider, some households had not sought treatment from biomedical providers. Of those who used herbs at home, 53.2% (*n* = 63) still used herbs from the first provider, five terminated at first provider and of the 28 that proceeded to the second provider, 14.3% (*n* = 28) went to churches/mosques for prayers. A total of 15 households out of 66 (22.7%) that provided either prayers or herbs at home had not accessed biomedical treatment by the second provider.

**Fig. 3:**
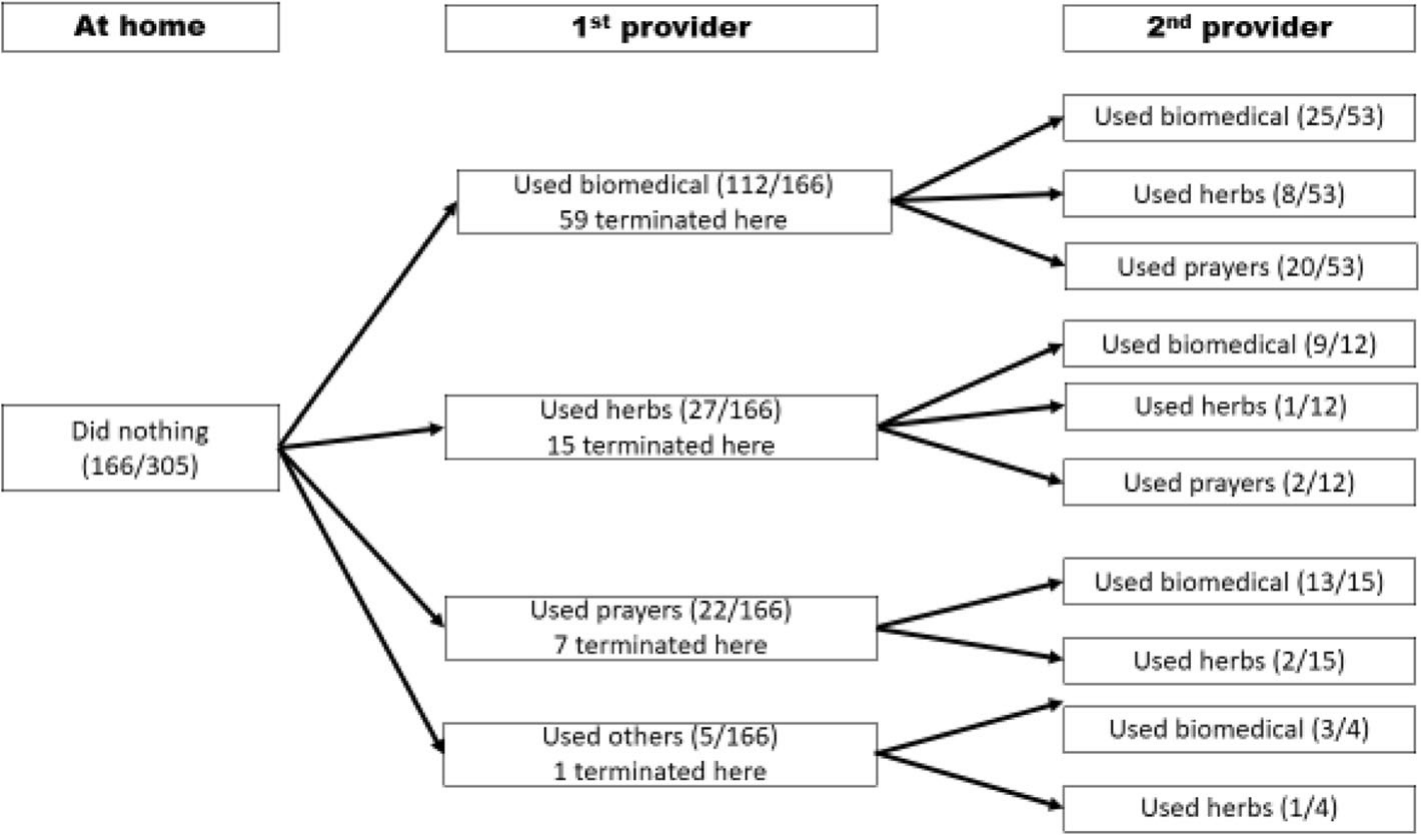
Treatment that those who did nothing at home received at first and second provider

An additional sub-analysis was then conducted for those who did nothing at home. This is presented in Fig. 3. Of the 166 households that did nothing, only 67.5% (112/166) went for biomedical treatment at first provider. However, there was another 16.3% (27/166) who went for herbs and 13.3% (22/166) that went for prayers. Of those who did nothing at home, 17.5% (29/166) had not received biomedical treatment for epilepsy by the second provider.

**Fig. 4:**
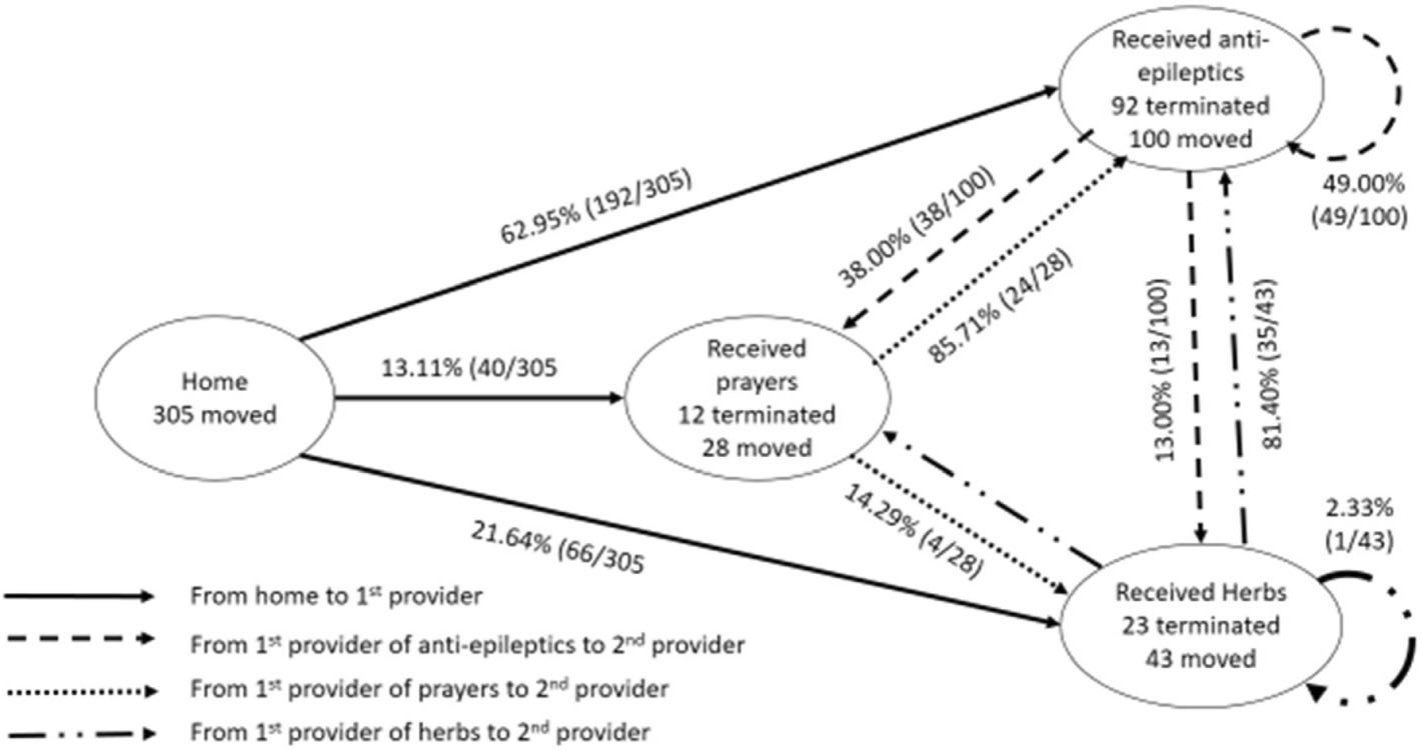
Treatment received at first and second provider

When households sought care outside the home, they received different treatment regimens depending on the provider. While health centres, hospitals, private clinics and drug shops would give anti-epileptic drugs like Phenobarbitone, Carbamazepine and Phenytoin with a few other drugs, churches and mosques offered prayers and traditional healers gave herbal drugs. Of the 12.8% (39/305) who sought care from the neighbour first, 34 got herbs, four received prayers and one got tablets for the treatment of malaria. The use of traditional herbs, modern medicine and prayers with the first and second care seeking encounter is shown in Fig. 4.

Of the 305 patients who moved out of the home, 63.0% (192/305) received anti-epileptics drugs, 13.1% (40/305) received prayers and 21.6% (66/305) received herbs. For the other seven patients, one did not recall where they went, one who went to the neighbour received church water, and five who went to health centres received other treatments but not anti-epileptic drugs. Of these seven, at the second provider level, the one who did not recall did not proceed to the second provider, five received anti-epileptics and one got herbal drugs.

### Bivariate analysis for choice of first health provider

Table 4 shows the unadjusted analysis comparing choice of health care seeking from a health centre to other sources such as hospital, church/mosque and friend/ neighbour. Other than a health centre, there was a significantly higher probability of attending a first choice provider at a hospital, if the respondent’s role in the household was that of a relative (Coeff 2.98, 95% CI 0.64, 5.31, *p* = 0.012) or the wife (Coeff 2.59, 95% CI 0.49, 4.70, *p* = 0.016), and was a government worker (Coeff 2.55, 95% CI 0.40, 4.70, *p* = 0.020), but was significantly less likely if the person was male (Coeff − 0.79, 95% CI -1.44, − 0.14, *p* = 0.017).

**Table 4:** Multinomial bivariate logistic regression showing association between choice of first provider and respondent’s characteristics

Variable	Hospital			Church/ Mosque			Friend/ Neighbour		
	Coeff	95% CI	p-value	Coeff	95% CI	p-value	Coeff	95% CI	p-value
Role in household									
Children	1			1			1		
Household head	1.96	−0.11, 4.03	0.064	−0.25	−1.40, 0.89	0.664	0.91	− 0.65, 2.46	0.253
Relative	2.98	0.64, 5.31	0.012*	−0.58	−2.96, 1.80	0.633	1.03	−1.18, 3.24	0.361
Wife	2.59	0.49, 4.70	0.016*	0.34	−0.90, 1.57	0.593	1.25	−0.38, 2.89	0.133
Age in completed years									
18–25	1			1			1		
26–35	0.97	−0.44, 2.38	0.178	1.62	0.02, 3.22	0.048*	0.77	−0.67, 2.21	0.295
36–45	1.69	0.32, 3.06	0.016*	1.23	−0.44, 2.90	0.149	0.83	−0.64, 2.30	0.270
46–55	1.57	0.18, 2.96	0.027*	0.99	−0.73, 2.72	0.258	0.88	−0.60, 2.35	0.244
56–65	1.23	−0.27, 2.73	0.107	0.66	−1.26, 2.58	0.503	0.76	−0.83, 2.35	0.348
> 65	1.44	−0.12, 2.99	0.071	0.99	−0.96, 2.94	0.318	1.28	−0.31, 2.87	0.114
Sex									
Female	1			1			1		
Male	−0.79	−1.44, −0.14	0.017*	−0.69	−1.48, 0.10	0.085	−0.81	−1.59, − 0.03	0.041
Marital status									
Married/ cohabiting	1			1			1		
Separated/ Divorced	0.38	−0.46, 1.22	0.372	0.10	−0.96, 1.15	0.860	0.18	−0.88, 1.24	0.737
Single/ Never married	−0.73	−1.91, 0.45	0.227	−0.53	−1.86, 0.80	0.436	−0.15	−1.37, 1.06	0.803
Widow/ Widower	0.04	−0.93, 1.01	0.934	−0.45	−1.79, 0.88	0.506	0.48	−0.56, 1.52	0.365
Highest level of education									
None	1			1			1		
Primary	−0.24	−1.03, 0.55	0.548	0.02	−0.96, 0.99	0.973	1.17	−0.11, 2.45	0.072
Secondary	0.14	−0.88, 1.17	0.787	0.23	−1.04, 1.50	0.725	0.67	−0.96, 2.31	0.421
Tertiary (Others)	1.37	−0.40, 3.14	0.130				1.99	−0.31, 4.29	0.090
Occupation									
Farming	1			1			1		
Government worker	2.55	0.40, 4.70	0.020*	1.35	−1.46, 4.15	0.347	2.57^&^	0.26, 4.88	0.029*
Trading	0.88	−0.14, 1.89	0.091	0.65	−0.63, 1.93	0.317	1.61	0.58, 2.64	0.002**
Unemployed	−0.34	−2.63, 1.95	0.770	1.35	−0.31, 3.01	0.112	1.39	−0.28, 3.05	0.102

Coefficients are relative probabilities compared to attending the health centre as first choice provider. *Significant at P < 0.05, **p < 0.01, ^&^Coefficient for bivariate analysis of choice of provider 1 as a traditional healer (not friend /neighbour), and occupation as predictor variable

Relative to attending a health centre as the first-choice provider, respondents were significantly more likely to attend a church/ mosque if they were aged 26 to 35 years (Coeff 1.62, 95%CI 0.02, 3.22, *p* = 0.048). Compared to attending a health centre as the first-choice provider, respondents who were traders were significantly more likely to consult a friend or neighbour (Coeff 1.61, 95%CI 0.58, 2.64 *p* = 0.002), but significantly less likely to consult a friend or neighbour if they were male (Coeff − 0.81, 95%CI -1.59, − 0.03 *p* = 0.041). Compared to attending a health centre as the first-choice provider, respondents were significantly more likely to choose a traditional healer (Coeff 2.57, 95%CI 0.26, 4.88 *p* = 0.029).

### Multivariable analysis for choice of first health provider

Table 5 shows the results for attending a first choice provider relative to a health centre, adjusted for the respondent’s background characteristics. Compared to attending a health centre as the first choice provider, the probability of opting for a hospital was significantly higher, if the hospital was perceived as nearer (adj.Coeff 2.16, 95%CI 0.74, 3.59, *p* = 0.003), or if respondents were traders by occupation (adj.Coeff 2.50, 95%CI 0.39, 4.61 *p* = 0.020).

**Table 5:** Multivariable multinomial logistic regression showing association between choice of first provider, and respondent’s characteristics

Variables	Hospital			Church/ Mosque			Friend/ Neighbour		
	Adj. Coeff	95% CI	*p*-value	Adj. Coeff	95% CI	*p*-value	Adj. Coeff	95% CI	*p*-value
Reason shifted from first provider									
Did not get better, hence sought better treatment	1			1			1		
Nearer	2.16	0.74, 3.59	0.003**						
Friend or neighbour’s advice	1.16	−1.42, 3.74	0.379	2.16	−0.45, 4.77	0.105	0.25	−2.31, 2.80	0.851
Age in completed years									
18–25	1			1			1		
26–35	2.51	−0.89, 5.90	0.148	2.54	−0.30, 5.38	0.079	1.45^&^	−2.64, 5.54	0.488
36–45	2.13	−1.16, 5.41	0.205	2.39	−0.52, 5.31	0.108	1.38^&^	−3.41, 6.17	0.573
46–55	2.82	−0.65, 6.30	0.111	2.80	−0.43, 6.03	0.090	5.83^&^	0.67, 10.99	0.027*
56–65	2.56	−1.05, 6.17	0.164	0.85	−2.84, 4.54	0.653	3.76^&^	−1.33, 8.86	0.147
> 65	2.16	−1.83, 6.16	0.289	0.84	−3.13, 4.82	0.678	6.56^&^	0.98, 12.13	0.021*
Sex									
Female	1			1			1		
Male	0.33	−1.98, 2.64	0.780	−2.01	−4.29, 0.28	0.085	−1.60	−3.77, 0.57	0.147
Marital status									
Married/ cohabiting	1			1			1		
Separated/ Divorced	0.69	−1.75, 3.13	0.578	−2.35	−5.50, 0.80	0.144			
Single/ Never married	0.98	−1.71, 3.66	0.475	−1.03	−4.10, 2.04	0.510	4.93^&^	0.56, 9.30	0.027*
Widow/ Widower	0.37	−2.43, 3.18	0.793	−1.34	−4.18, 1.49	0.353	−0.23	−3.38, 2.91	0.884
Occupation									
Farming	1			1			1		
Government worker									
Trading	2.50	0.39, 4.61	0.020*	1.29	−1.05, 3.63	0.281	2.87	0.71, 5.04	0.009**
Unemployed				1.66	−2.66, 5.97	0.451			
Currently receiving anti-epileptic medicines									
No	1			1			1		
Yes	−0.52	−1.86, 0.83	0.451	−0.22	−1.58, 1.13	0.746	0.57	−0.91, 2.05	0.452
Perceived purpose of treatment									
Control fits/seizures	1			1			1		
Cure epilepsy	0.71	−0.49, 1.92	0.247	1.91	0.38, 3.43	0.014*	1.29	−0.01, 2.58	0.051

Coefficients are relative probabilities compared to attending the health centre as first choice provider. *Significant at *P* < 0.05, **p < 0.01, ^&^Coefficient for bivariate analysis of choice of provider 1 as a traditional healer (not friend /neighbour), and occupation as predictor variable. Hosmer-Lemeshow goodness-of-fit Chi-square test is 30.51, df = 40, *p* = 0.860

Compared to attending a health centre as the first choice provider, the probability of opting for a church / mosque was significantly higher, if the respondent’s perceived purpose for their consultation was to get a cure for epilepsy (adj.Coeff 1.91, 95%CI 0.38, 3.48, *p* = 0.014).

Compared to attending a health centre as the first choice provider, the probability of opting for a friend or neighbour was significantly higher, and if their occupation was trading (adj.Coeff 2.87, 95%CI 0.71, 5.04, *p* = 0.009).

Also, compared to attending a health centre as the first choice provider, the probability of opting for a traditional healer was significantly higher, for the single or never married (adj.Coeff 4.93, 95%CI 0.56, 9.30, *p* = 0.027), for respondents aged 46–55 years (adj.Coeff 5.83, 95%CI 0.67, 10.99, p = 0.027), and those aged above 65 years (adj.Coeff 6.56, 95%CI 0.98, 12.13, *p* = 0.021).

## Discussion

Some patients with epilepsy go through many health providers as they seek treatment for an episode of epileptic attack although the majority terminate care seeking at the first and second providers. During the care seeking, patients use modern medicine, traditional herbs and prayers interchangeably.

Different health care providers are operating in the district. Each of the eight providers namely: health centres, hospitals, private clinics, drugs shops, churches/mosques, neighbours/friends, community outreaches and traditional healers received patients as first contact providers. Patients do not follow any clear direction as to who provides primary care for epilepsy and which provider offers secondary care. There is no clear referral system from one provider to the next. While some patients shift from a certain provider, others come to seek care from that same provider. A study in Eastern Uganda had noted the use of both biomedical and traditional healers in the management of child and adolescent mental health and the two sectors never referred patients to each other [12]. A study in Malawi among people with epilepsy indicated that they sought both traditional and modern medicine to treat their condition [13]. Other countries in Africa face this multiplicity of health providers like in Ghana [14] South Africa [15] and Ethiopia [16, 17]. It has been noted that biomedical treatment offered at health facilities is not adequate. There is lack of properly trained staff, capacity to diagnose epilepsy at many of these facilities, high cost and inadequate supply of drugs [1]. There is need to increase the capacity of biomedical services to provide appropriate treatment options for patients with epilepsy. This could be done through training of health workers and provision of adequate supplies to health facilities. When none of the providers gives an option that satisfies the patients or their relatives as being adequate management, patients continue rotating among different providers in search of appropriate treatment.

Respondents gave reasons why they moved from one provider to another with the majority saying they were seeking better treatment, more commonly reported than shorter distance to the next provider choice. When asked as to what they expected to get for the care seeking, patients at the first and second provider mostly indicated that they wanted complete cure of epilepsy. The services they received were not answering their needs, and they had inadequate knowledge of the outcomes of epilepsy considering the care they received. Various studies have highlighted the treatment gap in care for epilepsy patients [1, 2, 18, 19]. Low quality care for patients with epilepsy has been reported even in a study at the National Hospital in Uganda with more than half of the patients assessed having sub-optimal drug levels [5]. Addressing the human resource and drug supply across all the providers at the same time may not be feasible without huge resources to invest. However, since patients with epilepsy take drugs from home and they would need periodic appropriate assessment and a constant drug supply, instituting adequately staffed outreach places where patients can easily seek care would increase accessibility to appropriate treatment. The local authorities could strengthen certain known health facilities with specialized personnel and equipment to cater for the more complicated cases. It would help if an effective referral system is established between these outreaches and the central facilities with specialized care.

Despite the multiplicity of the care providers, the majority of the patients seek care from the health centres and hospitals which in this district majorly belong to the public sector. The private clinics and drug shops representing the private sector that offers western medicine in the treatment of epilepsy are reported to be catering for a small proportion of patients. A study in an urban resettlement in Northern India also found most patients with epilepsy seeking care from government facilities [20]. Epilepsy being a long term illness could be the factor driving patients to seek care in government facilities. Government facilities have been known for providing care to patients with chronic illnesses whereas private facilities take care for mostly those that need ambulatory care [21]. The median distance to public facilities in Uganda according to the National Service Delivery Report of 2015 is 3.2 km compared to only 1.2 km for private facilities [22]. The limited access of epileptic care from the private sector would mean that patients have to trek long distances to get treatment. This could be one of the reasons why patients shift to traditional herbalists who are usually close to the patients within the communities [23]. The Public-Private-Partnership policy framework stipulates that service delivery should utilize each sector’s comparative advantage [24]. Subsidized drugs for epilepsy could be distributed in the private sector like it has been done for malaria drugs and with adequate supervision, this would increase access and still maintain quality services [25].

This study highlights the use of prayers in churches and mosques, and other spiritual remedies like church water in the management of epilepsy especially for the younger age groups (< 35 years), and the use of traditional healers particularly the older age groups (> 45 years). The use of churches and mosques was associated with seeking cure for epilepsy. Many studies have indicated the use of traditional medicine and modern medicine [2–4]. Use of spiritual healing in sickness is documented in America and Asia [26–28]. During the epilepsy care seeking process in this study, many patients use different drugs for treating the same episode. There is a feeling of need for divine support in the healing process. Respondents in other studies in Uganda have attributed epilepsy to supernatural causes [4, 29]. People’s perception of disease influences their care seeking behaviour and which remedies they take. Community sensitizations need to be conducted in such communities to enlighten the communities on what treatment options to seek when their household members portray signs and symptoms of suspected epilepsy.

Non-adherence to treatment such as for a chronic condition as epilepsy is therefore affected by the increased financial burden associated with migration between epilepsy service providers, limited geographical access, and inadequate information as to the outcomes of treatment affecting treatment expectations. The results of this study highlight the importance of providing intensive counselling and adherence support particularly to younger epileptics (≤ 35 years) who might be naïve, and to the older epileptics (≥ 45 years) – who might develop treatment fatigue, as to the treatment expectations of a chronic condition such as epilepsy.

Community involvement came out prominently in the care seeking practices for epilepsy. A good proportion of the respondents indicated that their first place of seeking care was from neighbours or friends. It is also noteworthy that among the reasons people gave for moving from one provider to another was having been advised by a friend. Care seeking options have long been noted to be influenced by the significant others within the community [30, 31]. Communities need to be sensitized on appropriate care seeking since individual care seeking is partly influenced by the advice from community members. It is an endeavour that would need national support, district coordination and local level engagements. Community health workers (CHWs) as members of the local communities may help in changing community perceptions. Community health workers have been credited for spearheading community mobilizations and health promotion in child survival [32]. CHWs have improved engagement of depressed women with existing services [33]. A possibility of engaging CHWs as one of the mechanisms of changing community perceptions on epilepsy could be explored. Further, as the traditional healers are important stakeholders in the care for epilepsy, they could be targeted directly as a priority audience to be invited for community sensitisation about epilepsy care.

Results from this study should be interpreted in view of the study limitations we enlist. Firstly, we did not have a list of all households in the district which had members that were suffering from epilepsy (a sampling frame). Since all the public and private facilities are supposed to report to the district, the immediate source where we could get district wide information on villages with such households was the district office which supported the data collection. Due to this approach of enrolment of respondents, the potential for selection bias, due to over-, or under-reporting of households with epileptics by community health workers, could not have been avoided. Therefore a comprehensive survey for the entire district to generate data on all the households with epilepsy would be recommended. Secondly and relatedly, some respondents reported fever accompanying the seizures in some patients. It is possible that some of these may have been cases of febrile convulsions rather than epilepsy per se and it was not possible to rule them out as potential false positive cases misdiagnosed as epilepsy. A mitigating circumstance is that the people that identified these households are the community health workers who live within the communities and who among their activities include home and community management of fever. They are people who know and have witnessed children with febrile convulsions. In addition, febrile convulsions are not chronic illnesses. The community diagnosis for epilepsy is “a chronic (repetitive) seizure-like illness without fever”.

Thirdly, respondents were asked to recall the care seeking for the most recent episode. Some respondents may have had some loss of memory for the details. However, epilepsy is a chronic and traumatising illness in a family and events surrounding the care seeking especially the most recent episode would still be fresh in people’s memories. All our respondents were members of the households with the majority being the household heads who take a lead in at least financing the care seeking. The study had some strengths. It was done in more than 25% of the villages spread across all the sub-counties of the district. This made it possible to capture the different care seeking patterns that may be taken adopting to the availability of health facilities in the different sub-counties. Secondly, the study was done at household level and this helped to identify households who had not used the public health care system including those who had not received any biomedical treatment at all.

## Conclusion

Households in Masindi district navigate through many providers in seeking care for epilepsy. Although a big proportion access treatment from health centres and hospitals, there is a considerable proportion that do not even access biomedical treatment after visiting two providers. There is need to enable households with patients of epilepsy to access quality treatment. Some of the possible options could be working with CHWs to do health promotion focussing on awareness creation and community mobilization to promote positive health seeking behaviour. Community outreaches could be opened to treat patients nearer their homes. The private sector could be engaged to support treatment for epilepsy, such as through availing epileptic drugs for refill. Public facilities should be staffed with trained and skilled health workers as well as be stocked with adequate supplies so that patients can receive quality care and reduce on moving from one provider to another.

## Data Availability

As this data has village information which could identify respondent households, this dataset is not publicly available. However, data is available from the corresponding author upon reasonable request.
